# Penile fracture following masturbation: a case report

**DOI:** 10.1097/RC9.0000000000000557

**Published:** 2026-05-28

**Authors:** Fathallah Ibrahim, Al-Talep Ahmed, Alajrd Abd Alrhman, Al-Souqi Abd Al-Hadi, Alabed Yamen, Boubes Ebrahim

**Affiliations:** a Faculty of Medicine, Homs University, Homs, Syria; b Faculty of Medicine, Lattakia University, Lattakia, Syria; c Department of Urology, Al Mouwasat University Hospital, Damascus University, Damascus, Syria

**Keywords:** case report, management, masturbation, penile fracture, urological emergency

## Abstract

**Introduction and clinical importance:**

Penile fracture is an uncommon urological emergency caused primarily by direct trauma during intercourse. Patients usually present with acute pain, swelling, and deformity. Prompt clinical evaluation and surgical management are necessary to prevent long-term complications.

**Case presentation:**

A 22-year-old man presented with sudden pain, swelling, and loss of erection after his penis bent during masturbation. Examination revealed signs of a penile fracture but no urethral injury. Surgery identified a tear on the right side of the tunica albuginea, which was repaired. He recovered well and, after 1 year, had normal erections without pain or deformity.

**Clinical discussion:**

Penile fracture is a rare condition that occurs due to the rupture of the tunica albuginea following a strong injury during intercourse or masturbation. Histological changes within the tunica albuginea are associated with the traumatic rupture of the penis. For diagnosis, clinical examination and medical history are the primary tools, supported by imaging when needed. Surgery is the preferred treatment for most cases, as it allows rapid restoration of both structure and function.

**Conclusion:**

Penile fracture is a rare injury that requires quick diagnosis and surgical treatment to avoid complications. Early intervention and proper follow-up ensure the best functional and esthetic outcomes without long-term complications.

## Introduction

A penile fracture is an uncommon yet dangerous injury^[^1^]^. The most frequent cause of it is direct impact during intercourse, which results in an abrupt rise in cavernosal pressure and the subsequent rupture of the tunica albuginea^[^2^]^. Additional methods of injury include intense masturbation, forced bending of an erect penis, and unintentional trauma^[^3^]^. Although it varies by area and population, the incidence in the United States is roughly one case per 175 000 men. Developing nations continue to underreport it^[^1,4^]^. In one study, masturbation-related penile fractures accounted for 18% of all cases, while another study reported a similar rate of 19.8%^[^5^]^. Patients usually arrive clinically with a history of blunt trauma to an erect penis, which is followed right away by a snapping or popping sound, severe pain, and quick detumescence. Penile swelling, bruising, and a distinctive deformity known as the “eggplant deformity” are commonly observed upon physical examination^[^6^]^. While clinical evaluation is the primary method used to identify penile fractures, in more complicated instances, imaging techniques like magnetic resonance imaging (MRI) or ultrasound (US) can be used to assess the extent of the injury and rule out any related problems^[^7^]^. In the present case, a 22-year-old single male presented to the urology clinic with complaints of pain and swelling of the penis and was diagnosed with a penile fracture. This work is also reported in line with SCARE criteria, thereby increasing the report’s transparency and quality^[^8^]^.

HIGHLIGHTS

- Penile fracture is a rare emergency, and it is even more uncommon when it occurs during masturbation.
- Patients usually present with acute pain, swelling, deformity, and loss of erection.
- Diagnosis is mainly clinical, with imaging used when findings are uncertain.
- Early surgical repair provides the best functional and cosmetic outcomes.

## Case presentation

**Figure 1.**
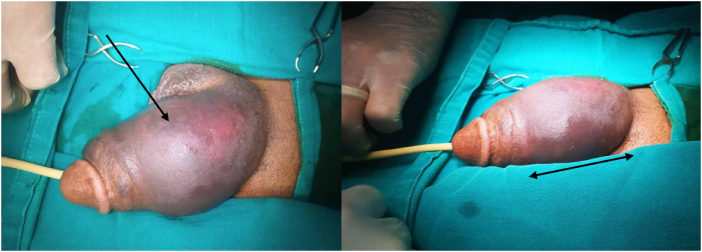
A preoperative image of the penis showing penile deformity with evident bruising and swelling (descriptive image of penile fracture). The urethral Foley catheter inserted prior to surgery is also visible.

**Figure 2.**
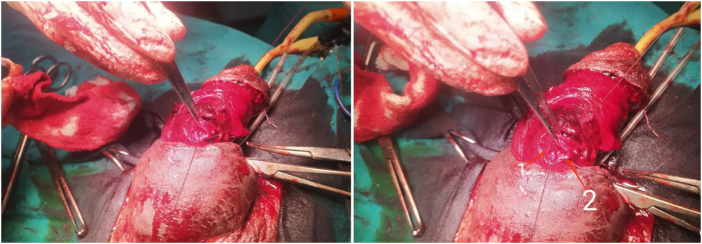
An intraoperative image, taken after the removal of the hematoma, shows the injury (tear) of the tunica albuginea on the lateral aspect of the right corpus cavernosum (arrow 1). The neurovascular bundle is clearly visible (arrow 2) adjacent to the lesion and was carefully preserved during the suturing and repair of the injury.

A 22-year-old single male presented to the urology clinic with pain and swelling in his penis that had started a few hours earlier. He said the pain began suddenly when his erect penis was accidentally bent during masturbation. He felt a sharp pain and heard a cracking sound immediately, after which the erection subsided, and the penis began to swell. There was no bleeding from the urethral opening, and he could still urinate without any difficulty. The patient was a young man with an unremarkable medical history, no prior surgeries, and no regular medications or drug allergies. He was single and reported no smoking, alcohol, or substance use. On examination, his penis was swollen and appeared deformed, primarily on the right side (Fig. 1). There was no blood at the meatus and no difficulty passing urine. His vital signs were all normal. Based on the history and physical examination, the diagnosis of penile fracture was made. The case was managed in a private hospital with limited diagnostic resources, where MRI was unavailable due to resource constraints. The medical team consisted of a consultant urologist, a surgical assistant, and a nurse. A urinary catheter was inserted to monitor his urine output, and antibiotics and pain medication were initiated. Subsequently, he was taken to the operating room. Under general anesthesia, the surgery was performed in the supine position. Skin preparation was carried out using povidone–iodine solution. We made a circumferential incision just below the glans and degloved the penis. There was a large hematoma under Buck’s fascia. Upon opening the fascia, we found a tear in the tunica albuginea on the right side at about the 9 o’clock position, near the neurovascular bundle. The tear was closed with interrupted 3-0 Vicryl sutures, a synthetic absorbable multifilament suture, providing adequate tissue support with gradual absorption over several weeks. The skin was closed in layers. The operation went smoothly without complications (Fig. 2). Postoperatively, the patient recovered well. He received antibiotics and painkillers, and the postoperative protocol included diazepam 5 mg for 5 days. Diazepam helps reduce anxiety and relax muscles, which can lower the chance of erections, decrease tension on the tunica albuginea sutures, and support healing. The catheter was removed on the second day after surgery. He passed urine normally without any pain or blood. On the third day, he felt comfortable, and we discharged him home in a clinically stable condition. To show the patient’s clinical course clearly, a brief timeline is presented in Table 1.

**Table 1:** Clinical timeline of the case.

Stage	Time	Description
Symptom onset	Night	The injury occurred during masturbation with sudden pain and a cracking sound
Presentation	10:00 AM	The patient presented to the hospital with pain and swelling
Evaluation and preparation	10:00 AM–1:00 PM	Clinical examination and preoperative preparation
Surgery	1:00 PM	Surgical repair performed successfully
Hospital discharge	Day 3	Patient discharged in stable condition
Follow-up	1 year	Regular follow-up visits with normal erections, no pain, no curvature, and excellent functional outcome

Postoperative wound care instructions included removing the dressing after 3 days and then changing it daily. We cleaned the wound with povidone–iodine and saline, and applied fusidic acid cream.

Sexual function scoring could not be used because the patient was single and had no sexual history. Therefore, the outcome was assessed clinically based on spontaneous and nocturnal erections. He was told to keep the wound clean and to avoid any sexual activity for about three months. Follow-up showed normal physiological erections without any curvature or associated pain. He said the wound had healed well and that his erections were back to normal. He did not complain of pain, curvature, or any other problems. No intraoperative or postoperative complications were recorded (Clavien–Dindo Grade 0).

## Discussion

Penile fracture is considered a rare urological emergency^[^2^]^. Penile fractures occur in middle-aged men, ranging from 30 to 50 years old^[^9^]^. The cause of this condition is the rupture of the tunica albuginea, the fibrous sheath surrounding the penis, where the rupture occurs due to a strong direct injury or trauma during vigorous intercourse^[^2^]^. This sheath can withstand pressure up to 1500 mmHg, but during erection, it becomes thin and thus susceptible to accidental rupture in the proximal part of the penis when subjected to abnormal bending, resulting in an excessive pressure increase in the corpora cavernosa^[^10^]^. Histological changes in the tunica albuginea are associated with traumatic rupture of the penis. Research studies have shown the presence of cellular infiltration and fibrosis in the ruptured tunica albuginea, suggesting a previous injury, which may result in a low rupture threshold compared to normal tissue^[^11^]^. Penile fractures vary in clinical manifestations, location, and severity. Injury to the tunica albuginea is the most common type, causing clinical symptoms including sudden pain, penile deformity, and swelling. A popping sound is heard during intercourse or trauma^[^12^]^. There are also many signs, such as blood at the urethral opening, pain during urination, or urinary retention, that appear when the rupture extends to the urethra, and this requires urgent evaluation to prevent complications such as urethral stricture^[^13^]^. Fractures can also be classified into unilateral fractures or bilateral fractures according to the injury of the corpora cavernosa. Bilateral fractures lead to noticeable deformity, which necessitates surgical intervention to reestablish function and appearance. In terms of diagnosis, understanding the types of fractures and symptoms is important for effective diagnosis and successful treatment^[^14^]^. In this case, a 22-year-old single man presented with penile pain and swelling for a few hours after bending his erect penis during masturbation, with loss of erection and a cracking sound. There were no signs of urethral injury. Penile fracture is diagnosed using physical exams, medical history, and imaging tests, but the clinical exam is the most important for confirming the diagnosis^[^15^]^. Because of its accessibility and capacity to identify abnormalities in the tunica albuginea, along with any related hemorrhage or injury to other structures, US is widely used as an initial imaging tool^[^7^]^. However, especially in complex instances, methods like computed tomography (CT) and MRI may yield more thorough information^[^16,17^]^. Sometimes, when there are worries about possible concomitant urethral or vascular damage, retrograde urethrography or cavernosography may be carried out^[^18^]^. There is debate between conservative treatment and surgery for penile fractures. Conservative care includes painkillers, antibiotics, and avoiding sexual activity, and it is often used when the diagnosis is unclear or surgery is not an option. Some studies show that it can work in selected mild cases without complications^[^19,20^]^. However, for the majority of penile fractures, surgical intervention is still the recommended course of action. A rapid anatomical and functional restoration, accurate defect repair, and direct visualization are all made possible by surgery, which also lowers long-term hazards. Concurrent problems such as urethral injuries or hematoma evacuation are also addressed^[^1,21^]^. Research supporting surgery demonstrates better results, such as decreased rates of erectile dysfunction, shorter hospital stays, and increased patient satisfaction^[^22^]^. Surgical complications can involve the formation of plaques and nodules, erectile dysfunction, scarring, painful erections, penile curvature or angulation, the need for additional surgery, urethral strictures, urinary problems, penile induration, and corporal aneurysms^[^2^]^. The literature review found only seven cases of recurrent penile fracture. In two cases, the injury was linked to sexual activity. Because this condition is very rare, it is hard to identify clear risk factors. However, aggressive sex and manual manipulation can increase the risk of it happening again^[^23^]^. In this case, early surgical treatment was performed with appropriate antibiotics and diazepam 5 mg for 5 days. For the best results, early detection and timely surgical intervention are essential^[^24^]^. According to studies, patients who arrive as soon as possible – within a few hours to a few days following the injury – have better results in terms of full symptom relief, erectile function maintenance, and cosmetic appearance^[^25^]^. Although the mechanism of injury is similar to that reported by Bhardwaj *et al* and both cases are rare, our case differs in several aspects. The tear was located at the 9 o’clock position near the neurovascular bundle, while in the reported case, it was a 1 cm rupture in the mid-shaft of the penis. In our case, the diagnosis was made clinically without imaging, unlike the other case where US was used, highlighting the importance of history and physical examination. The urinary catheter was removed on the second postoperative day compared to the third day in the reference case. Our follow-up was also longer, lasting 1 year versus 9 months, with good outcomes in both cases. We also used postoperative diazepam to reduce erections, which was not reported in the comparison case and may help reduce complications^[^10^]^.

## Strengths and Limitations

This case highlights a rare etiology of penile fracture and contributes to the limited literature on masturbation-associated injuries, but the report is based on a single case only.

## Conclusion

Penile fracture is a rare urological emergency, mainly based on clinical symptoms, although imaging can be helpful. Clinical examination remains the key step in diagnosis, while early surgical intervention is the best option to reduce complications. The use of postoperative diazepam may also help reduce erections and support healing.

## Data Availability

Data sharing is not applicable to this article, as no datasets were generated or analyzed during the current study.

## Patient perspective

The patient expressed satisfaction with the management and reported improvement in function and quality of life following the treatment.
